# Exploring the Immunological Aspects and Treatments of Recurrent Pregnancy Loss and Recurrent Implantation Failure

**DOI:** 10.3390/ijms26031295

**Published:** 2025-02-03

**Authors:** Jenny Valentina Garmendia, Claudia Valentina De Sanctis, Marián Hajdúch, Juan Bautista De Sanctis

**Affiliations:** 1 Institute of Molecular and Translational Medicine, Faculty of Medicine and Dentistry, Palacky University, 779 00 Olomouc, Czech Republic; jennyvalentina.garmendia01@upol.cz (J.V.G.); claudiavdesanctis@gmail.com (C.V.D.S.); marian.hajduch@upol.cz (M.H.); 2 Czech Advanced Technologies and Research Institute (CATRIN), Institute of Molecular and Translational Medicine, 779 00 Olomouc, Czech Republic; 3 Laboratory of Experimental Medicine, University Hospital Olomouc, 779 00 Olomouc, Czech Republic

**Keywords:** recurrent pregnancy loss, recurrent implantation failure, NK cells, T regulatory cells, Th17, Th1, Th2, macrophages, cytokines, HLA

## Abstract

Recurrent pregnancy loss (RPL) is defined as the occurrence of two or more consecutive pregnancy losses before 24 weeks of gestation. It affects 3–5% of women who are attempting to conceive. RPL can stem from a variety of causes and is frequently associated with psychological distress and a diminished quality of life. By contrast, recurrent implantation failure (RIF) refers to the inability to achieve a successful pregnancy after three or more high-quality embryo transfers or at least two instances of egg donation. RIF shares several causative factors with RPL. The immunological underpinnings of these conditions involve alterations in uterine NK cells, reductions in M2 macrophages and myeloid-derived suppressor cells, an increased Th1/Th2 ratio, a decreased Treg/Th17 ratio, the presence of shared ≥3 HLA alleles between partners, and autoimmune disorders. Various therapeutic approaches have been employed to address these immunological concerns, achieving varying degrees of success, although some therapies remain contentious within the medical community. This review intends to explore the immunological factors implicated in RPL and RIF and to analyze the immunological treatments employed for these conditions, which may include steroids, intravenous immunoglobulins, calcineurin inhibitors, anti-TNF antibodies, intralipid infusions, granulocyte colony-stimulating factor, and lymphocyte immunotherapy.

## 1. Introduction

Recurrent pregnancy loss (RPL) or recurrent spontaneous abortion (RSA) is defined as two or more consecutive pregnancy losses before 20 weeks (or 24) of gestation by the American College of Obstetrics and Gynecology and ESHRE Guideline Group on RPL and three or more losses by the World Health Organization [1,2,3,4]. It affects approximately 3–5% of women trying to conceive [1,2,3,4]. RPL can be primary, patients with no successful pregnancy, or secondary, unsuccessful pregnancies after a successful one [1,2,3,4]. The likelihood of a successful pregnancy depends on maternal age and the number of previous losses [1,2,3,4]. The pathophysiology of RPL is complex and involves maternal and fetal factors, possibly with more than one underlying factor [1]. There are many causes of RPL, including endocrine dysfunctions, uterine pathologies (uterus malformation, polyps, myomas, and adhesions), hydrosalpinx (accumulation of fluid in the fallopian tube), chromosomal abnormalities (quality of embryos), endometrial dysfunction, endometriosis, thrombophilia, chronic stress, high body mass index, male factor (sperm quality), infections, as well as immunological factors [5,6,7,8,9]. RPL, like other pregnancy disorders, is characterized by a loss of maternal–fetal immune tolerance [10]. The pathogenesis of RPL is unknown in almost 50% of women, and the condition is termed ‘idiopathic’ [11].

Recurrent implantation failure (RIF) refers to the unsuccessful implantation of three or more high-quality embryos or at least two egg donations [12]. Some causes of RIF are similar to those of RPL [12]. There are several risk factors for RIF, including advanced maternal age, smoking status of both parents, elevated body mass index, stress levels, vaginal microbiome dysbiosis, immunological factors (such as cytokine levels and autoantibodies), chronic endometritis (infection of the endometrium), hydrosalpinx, uterine polyps, myomas, congenital anatomical anomalies of the uterus, quality of sperm and embryos (genetic and epigenetic factors), endometrial receptivity, vitamin D deficiency, and genetic polymorphisms (HLA-G, p53, VEGF) [12,13,14,15,16,17,18,19]. miRNA and long-non-coding RNA (lcnRNA) have also been shown to be involved [20,21,22].

The immune system plays an essential role in normal implantation, maternal–placental fetal crosstalk, and embryo development [23]; thus, immunological alterations can be responsible for RPL and RIF. The local immune response can also be impaired by vaginal dysbiosis (VD). VB has been involved in several pregnancy complications, such as miscarriage, preterm birth, and adverse outcomes in vitro fertilization (IVF) [16,23,24,25,26]. A non-Lactobacillus-dominant microbiota in the endometrium was associated with reduced embryo implantation rate, pregnancy/continued pregnancy, and live birth rate [16,23,24,25,26]. 

It is important to note that RPL is linked to an increased risk of various medical conditions observed during pregnancy in those women who have conceived spontaneously [5]. These conditions include gestational diabetes, preeclampsia, placenta previa, placental abruption, miscarriage, preterm birth, cesarean section, perinatal death, and admission to the neonatal intensive care unit [27,28]. RPL is also a predictor of long-term cardiovascular disease and venous thromboembolism. Research has shown that patients with implantation failure have a significantly higher risk of early spontaneous abortion compared to those who have had successful implantations [29]. Despite the use of euploid blastocysts, the live birth rate per embryo transfer is generally reported to be around 50–60% [30]. 

RPL is also associated with psychological morbidity, poor quality of life of the affected couple, and a higher rate of marital problems [31] since it is highly frustrating to both couples and clinicians [32]. Psychological consequences of abortion are not exclusive to one of the partners and include increased anxiety, depression, post-traumatic stress disorder, and suicide [33,34]. This psychological condition may affect hormonal and circadian rhythms and the immune response [35]. 

This review explores the contribution of various immunological factors to RPL and RIF and discusses immunological interventions that may be employed in managing these conditions.

## 2. Innate Immune Response in RPL and RIF

The innate immune response functions as the initial line of defense against pathogens, encompassing mechanisms such as phagocytosis, endocytosis, secretion of lytic granules and protective peptides, and the release of proinflammatory cytokines, chemokines, lipid enzymes, metabolites, nitrogen, and oxygen radicals, all of which play crucial roles in the inflammatory process [36]. Furthermore, innate immunity contributes to tissue homeostasis and remodeling. The following section will describe and analyze the immune cells involved in pregnancy, as well as RIF and RPL. Table 1 presents a summary of different cells and immune responses. 

### 2.1. Natural Killer (NK) Cells

NK cells are large and granulate lymphocytes without antigen T cell receptor (TCR) or B cell receptor (BCR) [37,38]. There are two different types of NK cells: CD56dim/CD 16+ (pNK) and CD56bright/CD16−(uNK) [36]. pNKs are found in peripheral blood and are the most cytotoxic of the two, unlike uNKs, which are present in the uterus, produce more cytokines, and have regulatory functions [37,38]. Endometrial eNK and decidual dNK cells are also present [37]. The different subpopulations differ in their immune regulatory activity [37]. eNK cells constitute 30% of the total endometrial lymphocyte population before pregnancy, while dNK cells comprise up to 70% of the total decidual lymphocytes. dNK cells produce angiopoietin-2, placental growth factor, and vascular endothelial growth factor, expressing NKD2G, NKp44, NKp46, and NKp30 [37,38,39].

Abnormalities in NK cell activity were observed in most patients with RPL. Peripheral blood NK cell levels were significantly increased in women with RPL compared to controls [37,39,40]. Peripheral blood NK cell quantity was considerably higher in women with RIF (>18% of the lymphocyte count) than in fertile controls, with considerably activated NK cells (CD56dim/CD69+) [40,41]. In women with RPL, there are higher numbers of the cytotoxic CD56dim subtype and fewer CD56bright cells, even if the total cell population is unchanged [37,42]. The activation level of peripheral blood NK cells (CD69+) can predict pregnancy outcomes [43,44,45,46]. Type 1 cytokines such as IL-1, IL-2, and TNF-α increase the expression of CD16 on uNK cells and induce cytotoxicity against trophoblasts [47]. Both non-pregnant fertile and normal pregnant women had significantly lower NK cytotoxic responses, measured by flow cytometry at an effector-to-target cell ratio (E:T) of 50:1 compared to women with RPL and RIF [48]. 

Several studies have indicated an association between an increased population of uterine uNK cells and RPL and RIF [49,50]. A significantly higher frequency of endometrial CD56+ cells was reported in the mid-luteal phase of women with idiopathic RIF [49,50]. However, another study showed no correlation between uNK cell count and RPL pathology [51].

It is generally assumed that there is uncontrolled NK cell endometrial recruitment and/or failed CD56dim cell conversion to less cytotoxic CD56bright cells may occur in women with RPL [37,52]. However, a meta-analysis that evaluated uNK cells showed no significant difference in women with RPL compared to controls [53]. The CD16−CD56bright NK cell subset, predominant in the normal decidua and endometrium, was significantly decreased in favor of an essential contingent of CD16+CD56dim NK cells in RPL patients [54]. Notably, the percentages of CD56+ cells and CD16+CD56+ cells in the peripheral blood on the day of embryo transfer were significantly higher in the failed group than in the implanted group of infertile women who underwent IVF after intravenous immunoglobulin treatment [55]. In the endometrium, the increase in the percentage of CD16+CD56dim cells and the decrease in the percentage of CD16−CD56bright cells in the aborted group were significant compared to those of the delivered group [56]. Strobel et al. [57] showed that patients with secondary RPL had lower numbers of circulating CD56dimCD16brightNKG2D+ and CD56dimCD16brightNKp46+ than controls, suggesting that cytotoxicity receptors are also crucial in the process.

In non-pregnant women with idiopathic RPL or implantation failures, there was an increase in intracellular IFN-γ/TNF-α (defined as NK1 or inflammatory) and a decrease in IL-4/IL-10 (defined as NK2 or anti-inflammatory) in CD56bright pNK cells [58]. Pregnant women with recurrent miscarriages had a higher NK1/NK2 ratio, indicating a pro-inflammatory environment in the endometrium, which is detrimental to pregnancy [6,39]. Also, an increase in NK-CD8 expression (>60%) was predictive of IVF failure, while a decrease in expression (<40%) was significantly predictive of subsequent pregnancy failure [44]. Higher expression levels of NK-CD8+ were associated with elevated NK frequency, NK cytotoxicity levels, and CD158a expression in NK cells [44]. In women with RPL or implantation failure, the expression of natural cytotoxicity receptors (NKp46, NKp44, NKp30) and a2V-ATPase on CD56bright NK cells was significantly upregulated compared to that on CD56dim NK cells [58]. The differential expression of natural cytotoxicity receptors and a2V-ATPase in NK cell subsets may suggest dysregulation of NK cytotoxicity and cytokine production in women with RPL and implantation failure [58]. 

Proper interaction between maternal KIR and HLA class I, expressed by extravillous trophoblast cells, is crucial for the implantation and remodeling of uterine spiral arterioles [59]. Polymorphisms of KIR and HLA affect NK cell reactivity and susceptibility to recurrent miscarriage and preeclampsia. KIR 2DL2 expression was increased in RPL patients [60], and the association was stronger when there was an increased HLA-C2 allele frequency [61]. In a meta-analysis, KIR2DS2 and KIR2DS3 were significant risk factors for RPL, whereas the inhibitory gene KIR3DL1 was a protective factor [62]. A high frequency of KIR AA haplotypes that lacked activating KIR was found in women with RPL [63,64,65,66]. Moreover, patients with a KIR AA haplotype had significantly more risk of miscarriage if they underwent an IVF procedure compared to those who spontaneously achieved pregnancy [67]. The presence of HLA C2C2 in the fetus and the KIR AA haplotype in the mother correlated with implantation failure, recurrent miscarriage, and preeclampsia [62]. The balance of all activating and inhibiting signals between NK cells in the decidua and trophoblasts is an essential factor and may influence embryo implantation [67]. In another study, KIR A haplotype carriers experienced fewer pregnancy losses than KIR B haplotype carriers after euploid single-embryo transfer. However, this risk was modified when HLA-C alleles were present in the embryo. High-risk combinations (KIR A + homozygous C2 and KIR B + homozygous C1) resulted in a 51% increased risk of loss over all other combinations [67].

NKT cells, which express CD3 and CD56 markers, were increased in RPL [67,68,69]. Also, Tγδ cells may play a role in the process, as described by Xu and coworkers, 2021 [70]. These cells were shown to produce IL-10 upon stimulation with chorionic gonadotrophin Li et al., 2024 [71]. However, the roles of NKT and Tγδ cells in RIF and RPL are still unknown based on the complexity of the different possible subpopulations and the small number of circulating cells. More research is needed in this area.

### 2.2. Macrophages and Dendritic Cells

During pregnancy, macrophages and Treg cells maintain immune tolerance between the mother and fetus. Macrophages can change the decidual microenvironment in ways that contribute to RIF and RPL [72]. Two subpopulations of macrophages have been described: M1 (classically activated, induces inflammation and activates immunity) and M2 (alternatively activated, suppresses inflammation). M2 macrophages are abundant in the endometrium during the luteal phase and in healthy pregnancies. An M1/M2 macrophage ratio imbalance can lead to complications like preeclampsia, intrauterine growth restriction, RPL, and RIF [72,73,74,75,76]. In patients with unexplained RPL, macrophages in the decidua showed higher expression of CD80 and CD86 (costimulatory molecules) and lower expression of IL-10 compared to controls. Treg cells can inhibit the expression of CD80, CD86, and IFN-γ in macrophages while increasing the expression of IL-10 [76]. Macrophages (labeled with CD14) in the endometrium were significantly more abundant in patients with RPL than in controls [76,77,78,79]. In patients with RIF, the presence of diffuse adenomyosis (endometrial tissue in the myometrium) was associated with a marked increase in the density of macrophages and natural killer cells in the endometrial stroma compared to women with mild focal adenomyosis or no disease [79].

Dendritic cells (DC) play a crucial role in embryo implantation by regulating the immune response and aiding tissue remodeling [80,81]. They exhibit a tolerogenic phenotype and produce indoleamine 2,3-dioxygenase, which boosts the number of Treg cells while reducing Th1 cell survival and the cytotoxic activity of CD8+ T cells [80,81]. CD80/86 complexes on DCs in the uterus are downregulated and lead to the unresponsiveness of T cells, resulting in immune tolerance of the fetus. During implantation, artificial depletion of DCs or a high inflammatory milieu was associated with implantation failure [80,81]. A lower frequency of ILT4+ DCs was observed in the peripheral blood and endometrium of patients with RIF or RPL compared to the fertile control group [82]. Also, plasmacytoid dendritic cells were reduced in the decidual and peripheral blood of patients with RPL [83]. On the other hand, total DCs and myeloid DCs in peripheral blood were higher in patients with RPL than in controls [84]. In another study, there was no difference in peripheral DCs between RPL patients and controls in the first trimester of pregnancy [85].

Abnormal antigen presentation by DCs may not only lead to implantation failure and fetal rejection but also to the generation of autoimmune disorders. 

### 2.3. Polymorphonuclear Cells

Endometrial mast cells are essential components of tissular immune cells and play roles in endometrial tissue physiology and physiopathology [86,87]. Mast cells interact with macrophages in the female reproductive system [88]. Their presence was increased when RPL was activated [89] and was highly responsive to estrogen in endometriosis [90]. Since macrophage-colony stimulating factor 1 receptor (CSF1R) and mast/stem cell growth factor receptor KIT (KIT) are overexpressed in endometriotic lesions, treatment with pexidartinib, a tyrosine kinase inhibitor, was recently shown to decrease inflammation in endometrial tissue [91]. 

Eosinophils are scarcely present in the normal endometrium; however, they are present in endometriosis and involved in tissue repair and remodeling [92]. The migration of eosinophils is then due to an increase in eotaxin [93]. Chemokines are also present in the inflammatory profile of menstrual effluent [94], suggesting that eosinophils probably migrate to the tissue for a short time during the normal hormonal cycle. Their roles in RIF and RPL are not well known.

Neutrophils are absent in the normal endometrium except during menstruation [95]. However, they can be recruited under inflammatory conditions (infections, injuries), with increased chemokines and IL-17 affecting the endometrial tissue, hampering implantation, fetal survival, and preeclampsia/eclampsia [96].

### 2.4. T Cells

T lymphocytes are a crucial element of adaptive immunity. Both subpopulations, T helper (CD3+/CD4+) cells and T cytotoxic/suppressor (CD3+/CD8+) cells, play essential roles in fetal antigen recognition and modulation of local immunity [11]. The balance between Th1, Th2, and Th17 guides immune responses during pregnancy [37,39,42]. 

The proportion of CD8+ T lymphocytes in the endometrium was significantly reduced in patients with RPL, and the CD4+:CD8+ ratio was increased [54]. Conversely, the percentage of CD8+ T cells in peripheral blood was notably higher in women with RPL compared to the control group. The CD4+/CD8+ ratio was lower in women with RPL than in their healthy counterparts [97]. Furthermore, the total proportion of decidual effector memory CD8+ cells lacking PD-1 expression was elevated in cases of miscarriage [98].

Women with recurrent miscarriages had significantly higher absolute counts of central memory CD4+ T cells and CD8+DR+ T cells (activated cytotoxic cells) [99]. The frequency of NKG2D+ γδ T cells in lymphocytes was negatively correlated with the live birth rate in patients with RIF [100]. In a genetic study, the RIF group had a higher proportion of activated memory CD4+ T cells and a lower proportion of γδ T cells in the endometrium [101]. 

Women experiencing recurrent pregnancy loss (RPL) exhibited a higher frequency of the variable TCR beta (BV)-chain 19 of T cell receptors and a lower frequency of BV5.2 compared to the control group. This observation suggests that the specific skewed usage of TCR-BV may be associated with an increased susceptibility to RPL [102]. 

Regulatory T cells (Tregs), CD4+ CD25+ Foxp3+, have essential roles in the uterus, particularly during the peri-implantation period, and they are associated with the anti-inflammatory transition required for embryo receptivity [103]. Treg cells in the decidua and peripheral blood in unexplained RPL patients were statistically lower than those in control women, which may induce maternal lymphocyte activation to the fetal allograft [101,102,103]. Therefore, deficits in the number and/or function of Treg cells have been documented in cases of miscarriage and unexplained RPL [103,104,105,106,107]. Fewer Treg cells were associated with implantation failure [89,94] and had an altered phenotype in RPL and RIF [108,109,110]. Thus, CD4+ CD25+ Foxp3+ T regulatory cells may serve as a superior pregnancy marker for assessing miscarriage risk in pregnant women [111]. 

In decidual tissues from human miscarriage, the mRNA expression of CD28 was increased, while the expression of CTLA-4 mRNA (the checkpoint marker) was decreased. Therefore, the ratios of CTLA-4+/CD28+ in miscarriage were significantly lower than in normal pregnancy, both in peripheral blood and the decidua [112].

The balance between Th17 cells and Treg cells is believed to be crucial for pregnancy outcomes. Patients with unexplained RPL had higher levels of Th17 cells that secreted IL-17, GM-CSF, IL-21, and IL-22 in their peripheral blood and decidua [113,114]. There was a link between elevated Th17 cells and decreased CD4+ CD25+ Treg cells, which could contribute to developing unexplained RPL [104,109,110,111,113,114] and RIF [115,116]. The FoxP3/ RORγt ratio in fertile women was higher than in RIF patients [116]. 

Patients with RPL and positive anti-thyroid peroxidase (anti-TPO) antibodies showed a higher Th17 frequency than healthy control and anti-TPO+ control groups [117]. PD-1 + Th1 and PD-1 + Th17 cells were significantly lower in the RPL group than in controls, indicating a potential increase in Th1 and Th17 activity in women with RPL [118].

Wang and coworkers [119] reviewed the different T cell populations, including Th9, Th22, and T follicular cells (Tf), which were not discussed before. Th9 along with Th2 are essential to providing a tolerogenic milieu for the implantation phase [119]. Th22 protects trophoblasts from infections but also enhances trophoblast survival [119]. The role of Tf cells is partially known since regulatory Tf helpers have been proposed to aid in implantation and pregnancy [119]. Still, the roles of the other subpopulations are not well described in humans. In summary, the cytokines produced in the endometrial microenvironment during implantation and decidua formation are crucial for zygote survival. More research is required to understand the process of implantation. 

### 2.5. B Cells

The role of B lymphocytes in RPL has been less studied. B cells are believed to contribute to the success of pregnancy by decreasing the secretion of poly-reactive natural antibodies and producing protective blocking asymmetric antibodies [11]. A decrease in protective IgG maternal cytotoxic antibodies has been linked to RPL [120,121]. Additionally, anti-phospholipid antibodies were associated with RPL and preeclampsia [122]. Antibodies from women with RPL recognized specific endometrial antigens, which was not observed in normal multiparous women [121,122]. For more information, please refer to the autoimmunity section. On the other hand, B lymphocytes (CD20+) were increased in the endometrium of patients with RPL [55,123], and infertile patients had significantly decreased CD27+ B cells in their peripheral blood [123,124]. 

B cells have been associated with RPL [125]. However, the mechanism by which B cells are increased in the endometrium and peripheral blood of women with RPL is unknown. A decreased number of IL-10-positive B cells in the endometrial cavity has been related to RPL [125,126]. Even though IL-10 secretion in the endometrium may be protective for the fetus, there are still questions about the roles of B1 and B2 cells in normal pregnancy and RPL [125]. B1 cells are usually protective in tissues, producing IgM, while B2 cells are peripheral B cells that generate IgG and IgE antibodies [125]. The changes in B cell populations in the endometrial cavity may also be critical in producing deleterious antibodies to the fetus [125]. B cells may also present T cell antigens, generating an allogeneic response. In summary, many of the functions of B cells in the endometrial cavity are unknown; however, they may become an interesting pharmacological target to increase fertility and pregnancy success. 

### 2.6. Myeloid Suppressor Cells

Myeloid-derived suppressor cells (MDSCs) are a diverse group of cells of myeloid origin with an immature state and immunosuppressive function. There are two groups of MDSCs: monocytic MDSCs (M-MDSCs) expressing CD33+HLA-DR-/lowCD11b+CD14+CD15− and polymorphonuclear MDSCs (PMN-MDSCs) expressing CD33+HLA-DR−/lowCD11b+CD14−CD15+ [127]. MDSCs are increased in the uterus and peripheral blood during gestation [127]. In humans, PMN-MDSCs accumulate in the peripheral circulation of healthy pregnant women compared to non-pregnant controls [128,129]. In addition, in the peripheral blood of pregnant women, M-MDSCs are elevated [130]. On the contrary, in patients with RPL, MDSCs were reduced in the decidua and peripheral blood [128] and in the progesterone response [131]. In addition, patients with RIF showed significant reductions in blood PMN-MDSCs and M-MDSCs [132]. However, other authors found an increase of M-MDSCs in the peripheral blood of patients with RIF or RPL compared to controls and a negative correlation between M-MDSCs and Tregs in patients with RIF [132,133,134]. Screening by flow cytometry of these cell populations is not routine in RPL patients; however, it may be recommended to include the analysis of peripheral blood cells, such as T regulatory cells, Th17, and NK cells [134]. If possible, the presence of these cells should be confirmed in local tissue.

**Table 1:** Summary table of the different immune cells in RPL.

Cell Type	Physiological Function	Described Dysfunction in RPL	Ref.
Innate immunity	The response does not involve antigen presentation.		[36]
NK cells	Elimination of abnormal cells and pathogens. The tolerogenic response to fetus uterine and decidual NK cells differs from that of peripheral NK cells. Elimination of abnormal cells and pathogens.	Decrease in tolerogenic role and increase in cytotoxic response.	[37,38,39,40,41,42,43,44,45,46,47,48,49,50,51,52,53,54,55,56,57]
NKT cells	Elimination of abnormal cells and pathogens.	Increased cytotoxic function and involvement in local inflammation.	[67,69]
Tγδ cells	Control tissue homeostasis, phagocytosis of pathogens, and antigen presentation.	Increased cytotoxicity and involvement in local inflammation.	[70,71]
Macrophages	Present in the uterus. Involvement in tolerogenic responses.	Proinflammatory response and secretion of cytotoxic cytokines increase reactive oxygen and nitrogen species.	[72,73,74,75,76,77,78,79]
Dendritic cells	Efficient antigen presentation.	Abnormal antigen expression.	[80,81,82,83,84,85]
Mast cells	Present in the endometrium	Abnormal activation and proinflammatory role.	[86,87,88,89,90]
Eosinophils	Present in endometrium in part of the hormonal cycle.	Unknown.	[92,93]
Adaptative immunity	Requires antigen presentation. Highly selective.		[36]
T cytotoxic cells Th-1	Elimination of unwanted cells. Proinflammatory response. Activation of B cells. IgG production.	Involved in fetal rejection. Involved in fetal rejection.	[97,98,99] [101,102]
Th-2	Pro allergen response. Activation of B cells. IgE production.	Antagonism of Th1.	[101,102]
Th-17	Proinflammatory response.	Fetal rejection. Induces neutrophil migration.	[106,107]
T regulatory cells	Tolerogenic role to the fetus.	A decrease in these cells facilitates Th1 and cytotoxic functions.	[103,108,119]
B cells	B1 cells produce IgM against pathogens and protect tissue.	Decrease in B1 cells and increase in B2 cells in endometrium. Autoantibody production?	[121,122,123,124,125,126]
Myeloid suppressive cells	M-MDSC and PM-MDSC are involved in tissue tolerogenic response.	Impaired amounts of these cells in the endometrium.	[127,128,129,130,131,132,133,134]

## 3. Cytokines

Dysregulation of the interleukin network jeopardizes implantation, leading to RIF [135]. The overexpression of TNF-α and NF-κB also adversely affects implantation and leads to RIF. High ratios of IFN-γ/IL-4, IFN-γ/IL-10, and IFN-γ/TGF-β have been observed in RIF and are associated with adverse outcomes of implantation [135,136,137]. Th1-type (TNF-α, IFN-γ, IL-2) immunity to trophoblasts seems to be associated with unexplained recurrent abortion. It may play a role in reproductive failure, whereas T-helper 2 (Th2, IL-4, IL-5)-type immunity may be a natural response to trophoblasts, contributing to successful pregnancy [119,135,136,137,138,139,140]. In the peripheral blood and decidua of patients with RPL, the secretion of type-2 cytokines was decreased [141]. Similarly, the Th1/Th2 cytokine ratio was significantly higher in women with RIF than in healthy ones [138,142].

In previous studies, elevated Th17/Treg ratios were reported during the implantation window in patients with RPL [143,144,145]. We observed increased serum levels of IL-17 in patients with RPL compared to controls [105]. Additionally, high levels of IL-1β were observed in the uterine fluid of patients with RIF compared to fertile controls. At the same time, concentrations of IFN-γ and IL-10 were significantly lower [144,145,146]. Furthermore, IL-10 and TGF-β secretion was markedly lower in RIF patients, while IL-17 and IL-23 secretion was considerably higher in these patients than in controls [145]. Also, IL1-β, IL-6, IL-17, TNF-α, and the frequency of Th17 cells were increased in RIF patients with metabolic syndrome compared to RIF women without MS and the control group [146]. Endometrial stromal cells and whole endometrial cells of normal fertile women produced higher levels of IL-6, IL-8, and TGF-β than the RIF group. Additionally, endometrial stromal cells of normal fertile women produced lower levels of IL-10 compared to the RIF group [116,135].

Patients with RPL were found to have lower levels of IL-22 in the uterine decidua, which may contribute to a disruption in decidual homeostasis and ultimately lead to early pregnancy loss [147]. Similarly, the expression of IL-27 was lower in the deciduas of patients with RPL than in control subjects. IL-27 inhibited IL-17 expression and enhanced IL-10 expression in a dose-dependent manner [148]. Gene polymorphisms of IL-17 and IL-27 have also been associated with preeclampsia [149].

A study by Zhao et al. [150] found that serum IL-33 and soluble IL-33 receptor ST2 concentrations were higher in women with RPL. This suggests that these biomarkers could be used to predict and treat RPL. Additionally, research by Yue et al. [151] showed that levels of serum IL-35 were significantly lower in women with RPL compared to those in early normal pregnancy. 

Leukemia inhibitor factor (LIF) plays a vital role in various physiological processes during pregnancy, and its decrease was associated with RIF, as highlighted in a review by Mrozikiewicz et al. [17]. Similarly, LIF expression was altered in women with RPL, as reported by Karaer et al. [152].

A study conducted by Raghupathy et al. [153] demonstrated that ex vivo exposure to progesterone-induced blocking factor (PIBF) significantly increased the production of type 2 cytokines IL-4, IL-6, and IL-10 in lymphocytes from patients with RPL as compared with the production of IL-4 and IL-10 in lymphocytes from healthy pregnant women without affecting type 1 cytokine levels. PIBF decreased the type 1:type 2 cytokine ratio, indicating a shift toward a Th2 bias [153]. PIBF did not influence cytokine production in non-pregnant women, highlighting its role in inducing a type 1 to type 2 cytokine shift in pregnancy. Moreover, Kashyap and coworkers [154] showed that the levels of PIBF were reduced in women with RPL probably due to decreased transcription of progesterone receptor isoform B. The downregulation of receptors probably does not only affect the Th2/Th1 cytokine ratio but also can affect other immune cells, such as NK cells; more research is required in this topic.

Data analysis using PCR array found significantly higher expression of various cytokines and related factors (IL-6, IFN-γ, IL-17A, IL-23A, IFN-α1, IFN-β1, CD40 L, CCR4, CCR5, CCR6, CXR3, CCL2, IL-2, TLR4, IRF3, STAT3, RAG1, IFNAR1) in women with unexplained RIF compared to controls [155]. The study found lower expression of other factors (IL-1β, IL-8, NF-kB, HLA-A, HLA-E, CD80, CD40) in the unexplained RIF group compared to controls [155]. The authors concluded that the inflammatory responses based on pNK cells, the Th17 signaling pathway, and the TLR signaling pathway were activated in RIF [155]. Other factors may also be involved in the process since local secretion of cytokines involves not only stromal cells, lymphocytes, and epithelial cells. Moreover, the impact of the local microbiota can also affect cytokine secretion [26]. 

## 4. HLA in RPL and RIF

Haplotype analysis revealed that couples dealing with RPL had a significantly higher level of sharing MHC fragments among partners than control families [156,157,158,159]. In the Chinese population, the DQB1 × 0604/0605 allele may confer susceptibility to unexplained RPL, while the DQB1 × 0501/0502 allele may protect women from it [159]. Nevertheless, it was found that a high rate (3 or more) of HLA gene loci sharing (HLA-A, B, C, DR, DQ) in couples was associated with RIF [157,158,159,160,161,162].

Killing inhibitory receptors (KIRs) are critical in several pathologies. When the receptors bind the counterpart HLA ligand, a cytotoxic response may be decreased (L long KIRL) compared to others that may activate cells (S short KIRs) [163]. Decreased ligands for inhibitory KIRs could lead to insufficient inhibition of maternal uterine NK cells toward trophoblasts, thereby contributing to the pathogenesis of RPL [66,163,164]. The authors showed [164] that KIR 2DL2 (an inhibitory KIR)-positive Caucasian women with RPL and their partners had lower allele frequencies of HLA-C1 (the ligand for KIR2DL2) and a higher frequency of HLAC2 (ligand for another KIR receptor) as compared to KIR2DL2-negative women; thus, there was no KIR-related inhibition of cell killing [164,165]. These studies prompted the analysis of KIR genetics and their relationship with alloimmune reproductive failure. However, only a recent report has shed some light on the possible benefits of genetic screening [166]. 

The human leukocyte antigen G is a nonclassical HLA protein, displaying limited polymorphism, and is expressed in trophoblasts [167]. HLA-G has several splice variants (four membrane bound and three soluble isoforms) and immunomodulatory functions during pregnancy [167]. The HLA-G 14 bp insertion in the 3′UTR allele may increase the risk of RIF in Caucasians [168]. Soluble serum HLA-G (sHLA-G) levels were associated with RIF [169]. Patients carrying particular haplotypes differed in the secretion of sHLA-G [168]. A decrease in sHLA-G level after embryo transfer was observed when embryo transfer resulted in a lack of pregnancy [170].

While HLA analysis may not be at the forefront of research in RIF and RPL right now, it is an intriguing field brimming with unanswered questions just waiting to be explored. The potential for groundbreaking discoveries is immense.

## 5. Immune Checkpoints in RPL and RIF

Cell expression and soluble forms of immune checkpoint proteins in RPL and RIF have recently gained attention [171,172]. Not only PD-1/PD-L1/PDL2 but also OX-40/OX-40L, TIM-3, TGIT, and LAG-3 [171,172,173,174,175]. The rationale is that the expression of checkpoint inhibitors is related to tolerance in the implantation site, and their decreased expression is related to cell activation, consequent inflammation, and cytotoxicity. The expression of these markers in circulating lymphocytes or the assessment of soluble molecules could provide good biological markers for determining the severity of the disease and the effectiveness of the therapeutic response. 

## 6. Autoimmunity

Autoimmune diseases are characterized by immune system dysregulation, leading to humoral or cell-mediated immune responses against self-antigens. Several autoimmune diseases have been linked to RPL and RIF, particularly antiphospholipid syndrome, systemic lupus erythematosus, thyroid autoimmunity, and celiac disease. Furthermore, antinuclear antibodies, anti-thyroid peroxidase antibodies, and anti-phospholipid antibodies have been associated with recurrent pregnancy loss [176,177,178,179].

### 6.1. Antiphospholipid Antibodies (aPL) and Antiphospholipid Syndrome

Antiphospholipid syndrome is an autoimmune disease characterized by vascular thrombosis (venous or arterial) and/or pregnancy morbidity (pregnancy loss, fetal demise, premature birth before 34 weeks of gestation due to preeclampsia or placental insufficiency) associated with persistent antiphospholipid antibody positivity [180].

The presence of antiphospholipid antibodies, such as lupus anticoagulant (LAC) and anticardiolipin (aCL), has been closely linked to RPL [181,182,183,184,185]. The prevalence of aPL among women with RPL was about three times higher than that in fertile women [186]. Embryonic loss was more common in women with aCL IgM and women with double positive aPL (aCL + anti-β2-glycoprotein I or/and LAC). Clinical pregnancy loss was more common in women with positive anti-β2-glycoprotein I IgM. However, positive levels of aPL were rare in women with one or two prior pregnancy losses and were not associated with an increased rate of subsequent loss [187]. 

The presence of antiphospholipid antibodies (aPL) was linked to increased implantation failure after IVF, according to studies by Papadimitriou et al. [188] and Jarne-Borràs et al. [189]. However, a meta-analysis by Tan XF et al. [190] showed that although aPL positivity did not decrease the clinical pregnancy or live birth rate, it also did not increase the miscarriage rate in women undergoing IVF. The presence of aPL may inhibit the expression of LIF and homeobox A 10 (HOXA10) in the endometrium and influence pinopode development. This indicates that aPL positivity is associated with impaired endometrial receptivity, resulting in RIF, as found by Tan X and coworkers [191]. 

### 6.2. Systemic Lupus Erythematosus and Other Autoimmune Diseases 

Women diagnosed with systemic lupus erythematosus (SLE), pemphigus, scleroderma, undifferentiated connective tissue disease, and rheumatoid arthritis face an elevated risk of fetal loss [192]. Specifically, women with SLE exhibited an increased likelihood of experiencing various pregnancy-related complications, including but not limited to pregnancy loss, intrauterine fetal demise, preterm birth, fetal intrauterine growth restriction, and fetal congenital heart block [193,194]. In patients with SLE, diminished levels of complement C3 and C4 during the first trimester were correlated with a heightened risk of pregnancy loss. Notably, the risk of pregnancy loss may precede both the diagnosis and the manifestation of SLE [195].

Antinuclear antibodies (ANAs) penetrate cell membranes and produce cytotoxic effects. These effects are related to interrupting mitosis and damaging embryo quality, which can result in RIF [196]. The presence of ANAs in patients was correlated with an increased possibility of RIF after IVF, especially in older patients [197,198,199,200]. ANAs found in patients without defined autoimmune diseases increased the risk of RPL [179]. A meta-analysis showed a statistically significantly higher risk of RPL (more than threefold higher) in patients who were ANA-positive compared with those who were ANA-negative [196].

Primary Sjögren’s syndrome is responsible for an increased risk of spontaneous abortion [201]. Also, this disease is related to preterm delivery, congenital heart block, and pre-eclampsia [201,202]. 

A retrospective cohort study utilizing the TriNetX research network indicated that a prior diagnosis of RPL was linked to an increased risk of a subsequent diagnosis of an autoimmune condition, typically occurring between one and ten years following the diagnosis of RPL [203]. This study suggests a possible link between abnormal antigen presentation and RPL. 

### 6.3. Celiac Disease

Celiac patients, irrespective of their nutritional status (normal or under/overweight), presented a higher percentage of spontaneous abortions [204,205]. The incidence of idiopathic RPL doubled in patients suffering from celiac disease compared to healthy populations [204,205]. In a meta-analysis, the odds ratio value for celiac disease was 5.82 for women experiencing RPL [206]. Also, women with celiac disease had significantly higher risks of preterm birth, intrauterine growth restriction, stillbirth, low birth weight, and small for gestational age [207]. 

The pathogenic mechanisms that explain RPL in celiac disease could be nutrient deficiency (lack of elements like zinc, selenium, and folic acid) and the ability of anti-transglutaminase antibodies (which are present in celiac patients) to impair trophoblast invasiveness and increase their apoptosis and alteration of endometrial endothelial cell differentiation by inhibiting the activation of metalloprotease-2, disorganizing cytoskeleton fibers, and changing the physical and mechanical properties of cell membranes [208,209]. 

The HLA-DQ2/DQ8 polymorphism, which is associated with celiac disease, was more common in patients with RPL without a history of celiac disease than in control women without a history of miscarriage (52.6% vs. 26.6%) [210]. Patients with RPL and HLA-DQ2/DQ8 polymorphism had higher levels of anticardiolipin IgG and anti-peroxidase antibodies in comparison with patients with RPL without HLA-DQ2/DQ8 polymorphism [209]. Also, D’Ippolito et al. [210] found a statistically significant association between ANA and HLA DQ2/DQ8 positivity in women with RPL. Still, they did not observe a relationship between this polymorphism and positivity of anticardiolipin, anti-thyroglobulin, anti-thyroid peroxidase, anti-β2-glycoprotein, and anti-prothrombin antibodies [210,211].

### 6.4. Thyroid Autoimmunity

Thyroid autoimmunity, defined by the presence of autoantibodies against thyroid peroxidase and/or thyroglobulin (ATAs), is associated with RIF and RPL. This disorder causes thyroid function abnormalities and immune system imbalances [192,212]. ATAs can bind to the embryo’s surface and interfere with its development [212]. The cross-reactivity of ATAs with antigenic determinants of the egg, embryo, and placenta is another suggested mechanism leading to implantation and pregnancy complications [212]. Patients who test positive for ATAs exhibited significantly lower rates of fertilization, implantation, and pregnancy compared to those without these autoantibodies. In patients with RPL, the prevalence of anti-thyroglobulin antibodies was higher than in women without RPL [213]. In addition, the abortion rate was significantly higher in patients with ATAs [214,215]. The presence of ATAs may serve as a secondary marker for potential autoimmune disease rather than being the actual cause of pregnancy loss [214,215]. Moreover, increases in the population of endometrial T cells and INF-ɣ and decreases in IL-4 and IL-10 have been observed in women with autoimmune thyroid diseases who experienced reduced fertility and had ATAs compared to controls with no ATAs [216]. 

In two randomized controlled trials, the use of levothyroxine in euthyroid women with thyroid peroxidase antibodies did not result in a higher rate of live births compared to a placebo [217,218]. However, a clinical trial showed that treatment with levothyroxine decreased the risk of pregnancy loss and increased the live birth rate in RPL pregnant women who were positive for thyroid peroxidase antibodies or subclinical hypothyroidism [219]. Two metanalyses, one that included 787 infertile couples undergoing IVF/ICSI [220] and the other that contained fifteen eligible studies with 1911 participants [221], support the use of therapy in RPL.

To clarify the point, the guidelines of the European Society of Human Reproduction and Embryology (ESHRE) include hypothyroidism without and with autoimmunity [222]. Even though the evidence regarding the treatment effects of levothyroxine for women diagnosed with subclinical hypothyroidism and RPL remains inconclusive, medically defined hypothyroidism that occurs before conception or during the early stages of gestation should be managed with levothyroxine. While treatment for subclinical hypothyroidism may potentially decrease the risk of miscarriage, it is essential to weigh the possible benefits against the associated risks. In women with RPL and subclinical hypothyroidism who achieve a subsequent pregnancy, it is advisable to assess thyroid-stimulating hormone (TSH) levels during early gestation (between 7 and 9 weeks). Should hypothyroidism be confirmed, treatment with levothyroxine should be initiated. Nonetheless, women with thyroid autoimmunity and a history of RPL and TSH levels should also be evaluated during early gestation, and any identified hypothyroidism should be treated with levothyroxine. Conversely, euthyroid women who possess thyroid antibodies and have experienced RPL should not receive levothyroxine treatment.

More research is required to understand the importance of these autoantibodies in RIF and RPL.

## 7. MicroRNAs (miRNAs) and RPL

MicroRNAs (miRNAs) affect immune cell differentiation, proliferation, and function [223]. They are short, non-coding RNAs, typically 22–24 nucleotides in length, that regulate protein production by inhibiting mRNA translation or inducing mRNA degradation through binding to the 3′ untranslated region of mRNA (UTR) [223]. They play critical roles in differentiating T helper cells and developing Treg cells [223,224,225]; thus, the balance of miRNA is crucial for both cells in RPL and RIF.

The dysregulation of miRNA expression is associated with RPL [226,227,228]. In a recent review, 75 different miRNAs showed a significant difference in expression between women with RPL and the control group. In total, 53.33% of these miRNAs had increased expression, 28% had decreased expression, and 18.66% had both increased and decreased expression, depending on the study [227]. In a study using plasma samples, 77 miRNAs were upregulated and 31 were downregulated in the RPL group compared with the regular pregnancy group [228].

In women who had experienced miscarriages but had normal karyotypes, there was a noted overexpression of miRNA-133a [229]. This overexpression may lead to a reduction in HLA-G protein expression [229]. This reduction may affect the protection of the fetus from possible aggression from immune cells [229]. Additionally, miR-30e, miR-34a-3p/5p, miR-141-3p/5p, miR-24, miR-486-3p, miR-6126, and miR-6754-3p were found to be dysregulated in the decidual natural killer (dNK) and peripheral natural killer (pNK) cells of RPL patients [230]. 

Specific single nucleotide polymorphisms (SNPs), such as miR-21 rs1292037 and miR-155-5p rs767649, have been linked to higher rates of RPL [230]. However, only one report exists, and it needs to be confirmed.

Twelve differentially expressed miRNAs were identified in the sperm of male partners of idiopathic RPL patients; eight miRNAs (hsa-miR-4454, hsa-miR-142-3p, hsa-miR-145-5p, hsa-miR-1290, hsa-miR-1246, hsa-miR-7977, hsa-miR-449c-5p, and hsa-miR-92b-3p) were upregulated and four (hsa-miR-29c-3p, hsa-miR-30b-5p, hsa-miR-519a-2-5p, and hsa-miR-520b-5p) were downregulated [231].

This topic is relatively new, and there is still room for improvement; the roles of extracellular vesicles and the modulation of different types of RNA in aging and senescence can be crucial for implantation and fetus survival [232].

## 8. Microbiota in RPL and RIF

Despite uncertainty in the causal relationship between the endometrial microbiota and early pregnancy loss, there is some evidence that the endometrial microbiota may be predictive of RPL [233]. RIF and RPL are associated with increased microbiome diversity and a loss of Lactobacillus dominance in the lower female reproductive system [26,233,234,235]. First-trimester miscarriage has been associated with a reduced prevalence of *Lactobacillus* spp., which dominates the normal vaginal microbiota [236]. A vaginal microbiota depleted of Lactobacillus spp. was related to pro-inflammatory cytokine (IL-1β, IL-6, IL-8) levels most strongly in euploid miscarriage compared to viable term pregnancy [237].

In a study by Peuranpää et al. [238], it was found that *Lactobacillus crispatus* was less abundant in the endometrial samples of women with RPL compared to the control group. Additionally, *Gardnerella vaginalis* was found to be more abundant in the RPL group than in the controls in both endometrial and vaginal samples. Furthermore, Vomstein et al. [239] observed a lower abundance of *Lactobacillaceae* in RPL and RIF patients at three points of the menstrual cycle. They found increases in *Proteobacteria* in the RPL and RIF groups toward the end of the menstrual cycle [239]. On the other hand, the RIF group exhibited a remarkably diverse composition, unlike the control and RPL groups [240].

The presence of a non-Lactobacillus-dominated endometrial microbiota, <90% *Lactobacillus* spp. and >10% of other bacterial taxa, in a receptive endometrium has been associated with significant decreases in the rates of implantation, pregnancy, ongoing pregnancy, and live birth among infertile patients undergoing in IVF [240]. The increased abundances of specific taxa—such as *Gardnerella, Haemophilus, Klebsiella, Neisseria, Staphylococcus, Streptococcus, Atopobium, Bifidobacterium,* and *Chryseobacterium*—in endometrial samples were linked to instances of abortion or absence of pregnancy [240].

A relative dominance of *Ureaplasma* species in the endometrial microbiome was an independent risk factor for subsequent miscarriage with normal karyotype in a cohort of patients with a history of RPL [241]. *Proteobacteria* and *Firmicutes* were significantly elevated in RPL patients compared to women requesting termination of normal pregnancy [239,241]. On the other hand, the abundances of *Bacteroides* and *Helicobacter* in the vagina in the early embryonic arrest group were higher than in the normal pregnancy group, and the abundance of *Lactobacilli* in the normal pregnancy group was higher than in the embryonic arrest group. In this last group, the abundance of *Lactobacillus inners* was significantly lower than in the normal pregnancy group [242].

In light of the compelling evidence presented, it is recommended that screening for the vaginal and endometrial microbiota, as well as for papillomavirus, which influences the local microbiota, be conducted routinely in patients who are preparing to undergo medical treatment for infertility and IVF.

## 9. Immunological Treatment of RPL and RIF

Different treatments have been used in both RIF and RPL. Table 2 summarizes the various therapies found in the literature. The American Society of Reproductive Medicine (ASRM) and the ESHRE have published guidelines based on the evidence found in the literature, and the recommendations are based on strong or weak proof of the therapy analyzed [222,243]. The ASRM only validates the use of heparin and aspirin in patients with antiphospholipid syndrome. The society does not recommend any specific treatment for other RPL cases. On the other hand, the ESHRE has some recommendations that will be discussed at the end. 

Table 2 summarizes the therapeutic approaches discussed in the literature to provide the reader with a comprehensive background on the subject. 

### 9.1. Corticosteroids

Prednisolone is beneficial for women who have experienced miscarriage and have increased numbers of NK cells. This steroid reduces the frequency and function of NK cells [122,244,245]. However, one study reported a live birth rate of 60% with prednisolone compared to 40% with a placebo, but this difference was not statistically significant [246]. The difference may be related to Tang and coworkers’ general screening and follow-up [246].

Prednisolone also improved implantation in IVF patients with high peripheral CD69+ NK cells [247]. In a retrospective study involving RPL and RIF, prednisolone significantly reduced uterine NK cells, although normalization was achieved in only 48.3% of patients [248]. Furthermore, there was no significant difference in pregnancy outcomes or complications between women who received prednisolone and those who did not [248]. However, a meta-analysis provided evidence that prednisolone therapy improved pregnancy outcomes in women with RPL [249]. Likewise, a network meta-analysis found that aspirin combined with glucocorticoids improved the miscarriage rate in patients with RIF [250]. Patients with RIF prednisone treatment had increased Treg cells and an improved Th17/Treg ratio [251,252,253].

In a randomized controlled trial, the fertilization, pregnancy, and implantation rates were significantly higher in patients with RIF and positive for antinuclear antibodies treated with prednisone (10 mg/day) and aspirin (100 mg/day). In comparison, the abortion rate was markedly higher in the non-treatment group [254]. Low-dose corticosteroids was effective for autoantibody (antinuclear antibodies, anti-DNA, or lupus anticoagulant)-positive women with RIF, reaching significant increases in pregnancy and implantation rates [254]. However, among patients with recurrent implantation failure without autoimmunity, treatment with prednisone did not improve the live birth rate compared to a placebo, and the use of prednisone may have increased the risk of preterm delivery and biochemical pregnancy loss [255].

In refractory antiphospholipid antibody-related pregnancy loss, using first-trimester low-dose prednisolone (10 mg/day) alongside conventional treatments such as aspirin and heparin may be beneficial [256,257]. For patients with previous IVF failure and significant serum anti-ovarian antibody levels, prednisolone (0.5 mg/kg) has been shown to improve pregnancy, implantation, and live birth rates [258]. However, it is essential to be aware of the potential side effects of steroids, which include insomnia, increased appetite, headache, palpitations, hirsutism, nausea, and mood alterations [246,247]. Furthermore, the use of steroids can increase the risk of gestational diabetes mellitus, preeclampsia, preterm birth, and low birth weight [259,260].

### 9.2. Hydroxychloroquine

Hydroxychloroquine has been found to have anti-thrombotic, anti-inflammatory, and immunomodulatory properties [261]. Studies have shown that in patients with antiphospholipid syndrome (APS) and miscarriage, the addition of hydroxychloroquine to conventional treatment improved live birth rates and reduced pregnancy loss [261,262,263,264,265]. It was observed that the effects of hydroxychloroquine on the live birth rate were dose dependent, with the best rate at 400 mg daily (94%) compared to 200 mg daily (79.5%) in patients with refractory APS [265]. Furthermore, patients with persistent positivity for aPL antibodies who received hydroxychloroquine (200–400 mg/day) had reduced adverse pregnancy outcomes, especially fetal loss at >10 weeks of gestation and placenta-mediated complications such as preeclampsia, placental abruption, and intrauterine growth retardation [266,267].

In RIF women, hydroxychloroquine enhanced Tregs and diminished Th17 responses. However, it did not improve pregnancy outcomes [268]. In a recent non-randomized study, exposure to hydroxychloroquine in early pregnancy for women with a history of RPL did not seem to prevent further miscarriages [269].

There is still room for improvement in corticosteroid therapy in women with an autoimmunity spectrum.

### 9.3. Calcineurin Inhibitors 

Calcineurin inhibitors are a group of immunosuppressive agents that specifically inhibit calcium/calmodulin-dependent phosphatase calcineurin in blocking T cell activation, cytotoxicity, B cell growth, and antibody production. Tacrolimus and cyclosporine A are calcineurin inhibitors. Tacrolimus binds to FK-binding protein-12 to produce a complex that inhibits calcineurin, while cyclosporine A binds to cyclophilin to generate a complex that does the same [270]. These drugs have not been associated with an increased risk of birth defects [271]. 

In a meta-analysis study, treatment with calcineurin inhibitors (cyclosporine and tacrolimus) in patients with RPL or RIF increased the live birth rate and clinical pregnancy rate and decreased the miscarriage rate compared to the control group [272,273]. Treatment with low-dose cyclosporine A (100 mg or 150 mg/day for 30 days or 6 months, initiated after a positive pregnancy test) increased the live birth rate in RPL patients and reduced the miscarriage rate [274,275]. In the cyclosporine group, there were significant decreases in Th1 frequency, Th1/Th2 ratio, T-bet mRNA expression (Th1 marker), INF-γ (Th1 cytokine), and TNF-α (Th1 cytokine). Moreover, there were significant increases in Th2 frequency, mRNA expression of GATA binding protein 3 (Th2 marker), and IL-10 secretion in the cyclosporine group [275]. In addition, in a nonrandomized trial, cyclosporine achieved a 77% live birth rate in RPL patients who did not respond to other therapies (aspirin, prednisone, heparin, and immunotherapy with their husband’s mononuclear cells). However, a significant group of patients had hypertensive disorders (without symptoms of preeclampsia) and preterm delivery [276]. On the contrary, for patients with RIF not selected by immunological profile, there was no difference in the adjusted odds ratios of implantation, clinical pregnancy, chemical pregnancy, take-home baby, and multiple births rates, preterm birth, abnormal birth weight, or sex ratio between the cyclosporine-treated group (150 mg/day for 2 weeks) and the control group [277].

It has been found that using tacrolimus in low doses improved pregnancy outcomes for women with immune disorders and RPL [278,279]. Tacrolimus was more effective than a placebo in reducing pregnancy complications [278,279,280]. Additionally, tacrolimus has been found to improve reproductive outcomes in women with repeated implantation failure and elevated peripheral blood TH1/TH2 cell ratios [281]. In another study, treating RPL women with high TH1/TH2 cell ratios with vitamin D and tacrolimus resulted in significantly higher clinical pregnancy and live birth rates [282]. Furthermore, the combination of tacrolimus and low-molecular-weight heparin improved pregnancy outcomes for patients with elevated peripheral NK cells [281]. It has also been noted that using cyclosporine and tacrolimus in low doses and for a short time appears safe. It does not lead to serious side effects nor increase the risks of obstetric and neonatal complications [281,282,283,284].

Sirolimus (rapamycin) is an mTOR (mammalian target of rapamycin) inhibitor and autophagy inducer. mTOR is a serine/threonine kinase that plays a regulatory role in cell metabolism, proliferation, and differentiation, while autophagy is a process involved in the decomposition and recycling of cells [285]. It degrades proteins, organelles, and extracellular invasive substances during cell stress and lack of nutrition. Autophagy is involved in endometrial decidualization and trophoblast invasion, and mTOR can inhibit the autophagy process [285]. Sirolimus may reduce the risk of miscarriage by enhancing endometrial and macrophage autophagy. However, this medication could be deleterious to pregnancy [285,286]. Also, sirolimus may reduce the occurrence of RPL and RIF by reversing abnormality of the mTOR/autophagy axis and regulating immunity [285,286].

In a double-blind, phase II randomized clinical trial, sirolimus treatment (2 mg/day for 17 days) increased Treg cell number and function in the treated group of patients with RIF and altered the Th17/Treg ratio. Moreover, there was a higher clinical pregnancy rate (55.81%) in sirolimus-treated patients compared to controls (24.24%) and an increased live birth rate (48.83%) in RIF women who received sirolimus compared to controls (21.21%) [285]. Since this study refers to a clinical trial published in 2019, it is not easy to envision that the drug is safe to use in complex cases of RIF and RPL.

### 9.4. Intravenous Immunoglobulins (IVIGs)

IVIGs have multiple mechanisms of action. They reduce the activity of NK cells, increase the activity of Treg cells, block anti-HLA antibodies, prevent complement activation, downregulate stimulatory Fc receptors (FcγRI and FcγRIII), and upregulate inhibitory receptors (FcγRIIB) on the surface of different immune cells [286,287,288,289]. IVIGs can significantly increase the live birth rate in RPL. Higher doses of IVIG in the presence of autoimmunity tended to increase the success rate of pregnancy. However, more high-quality randomized controlled trials, suitable for different populations, races, dosages, and timings of IVIGs in the treatment of recurrent abortion, are needed to confirm their effectiveness [290,291,292,293,294]. Administration of IVIGs at a dose of 400 mg/kg per treatment spaced every 3 to 4 weeks is likely to have clinical efficacy in women with RPL and cellular immune abnormality [290,291,292,293,294]. In a retrospective study, IVIGs at a dose of 600–800 mg/kg before conception and monthly during pregnancy until 16–20 weeks of gestation were associated with a higher live birth rate, especially in those with five or more abortions and primary RPL [295]. A retrospective study involving RPL patients found that administering IVIG at a dose of 200 mg/kg every 2 to 3 weeks during the first trimester, followed by monthly doses until the end of the second trimester, in conjunction with low-dose aspirin treatment, resulted in a live birth rate of 73.5% [296]. Additionally, this study found no significant correlation between NK cell counts and the live birth rate [296]. In RPL patients, substantial reductions in Th1 lymphocyte frequency, transcription factor expression, and cytokine levels were observed in the IVIG-treated group with an increment of NK cells. The Th1/Th2 ratio decreased significantly after treatment, and 87.5% of women in the IVIG-treated group had live births compared to 41.6% of the untreated group [296].

IVIGs might be more effective in a subgroup of women with an aberrant immunological profile. The effect of IVIGs was particularly marked in a subset of studies that included patients based on the presence of elevated NK-cell percentage (>12%) and the time of intervention (before or during the cycle of conception). Treatment with IVIGs may improve live birth rates in women with RPL and underlying immune conditions. However, these results should be interpreted cautiously as studies are limited by a low number of participants and non-randomized designs [296].

In patients with RIF, the use of IVIGs was associated with a higher implantation rate compared to a placebo. Clinical pregnancy and live birth rates were significantly increased in patients randomized to IVIGs. Moreover, the miscarriage rate was significantly lower in patients randomized to IVIGs [297,298,299,300]. The administration of IVIGs has been indicated to reduce the Th1/Th2 ratio and effectively boost the reproductive outcome of RIF patients with high Th1/Th2 ratios or low Treg/Th17 ratios [299,300,301]. Furthermore, IVIGs downregulated the Th17 cell population and upregulated the Treg cell population in women with RPL [300]. In addition, IVIGs decreased NK cell levels and cytotoxicity in patients with RPL or RIF [301,302]. In one study, the live birth rate was significantly higher when IVIGs were administered before conception but not after implantation [303]. Therefore, pre-conception treatment with IVIGs should be considered in women with RIF of immune etiologies [303]. A meta-analysis found that IVIG treatment was associated with a relative risk (RR) for a live birth rate of 1.26 in secondary RPL versus an RR of 0.88 in primary RPL [303]. 

In subfertile women with high preconception Th1/Th2 ratios and/or an increase in NK cells (CD56+/CD16+), the IVF success rate significantly improved after IVIG therapy compared to no treatment [304,305]. In patients with typical Th1/Th2 ratios and normal CD56+ cell levels, IVF success rates were no further improved with IVIG therapy [304,305]. Therefore, IVIGs may be helpful for patients with previous IVF failure and elevated preconception Th1/Th2 ratios and/or NK cells [304,305]. In a meta-analysis of patients with RPL or RIF and elevated NK cells, the results pooled from IVIG studies, which included 557 women (312 interventions and 245 controls), showed a risk ratio favoring the group that received intervention; however, there was significant heterogeneity and a moderate to severe risk of bias in the included studies [306]. Nevertheless, a Cochrane review reported no significant effect of IVIGs on live birth rates in patients with RPL [307]; several authors doubt the report [293,294,295,296,297,298,299,300,301,302,303,304,305,306]. 

In a recent double-blind, randomized, placebo-controlled trial in patients with four or more RPL and unknown risk factors, the IVIG group had a higher live birth rate (58.0%) than the placebo group (34.7%). In this trial, high doses of IVIGs (400 mg/kg/day for 5 days) increased Treg cells and decreased natural killer cell activity [308]. IVIGs are an effective and safe treatment for pregnant patients affected with SLE and RPL [302]. IVIGs have been used in patients with antiphospholipid syndrome and a history of stillbirth, plus low doses of aspirin, low-molecular-weight heparin, hydroxychloroquine, and prednisone, with good results [309,310]. Couples with recurrent IVF failure and HLA similarity (at least 3 HLA loci) may benefit from IVIG treatment [310].

Despite the number of reports favoring IVIG treatment in RIF and RPL, some patients still do not respond to treatment, and it is challenging to determine the causes of such an effect. On the other hand, the decrease in blood products may further affect the possibility of treatment in these complicated patients. 

### 9.5. Granulocyte Colony-Stimulating Factor (G-CSF)

G-CSF can increase IL-10 synthesis by Treg cells and promote transplantation tolerance, thereby improving endometrial remodeling and receptivity [311,312]. In a randomized controlled trial, 82.8% of women with RPL treated with subcutaneous G-CSF delivered a healthy baby, compared to 48.5% for the placebo group (*p* = 0.006) [313]. However, in another randomized controlled trial, there was no difference in the live birth rate between women with RPL and G-CSF treatment and women on a placebo [314].

In a meta-analysis, subcutaneous G-CSF administration was beneficial for clinical pregnancy rates in women with RIF [315]. Furthermore, in the RIF population, administration of G-CSF was associated with a significantly higher clinical pregnancy rate than no intervention [316]. On the contrary, a single dose of subcutaneous G-CSF 30 min before embryo transfer in patients with RIF induced no significant differences compared to controls in the abortion rate, clinical pregnancy rate, or live birth rate [317]. In another meta-analysis, subcutaneous G-CSF was more effective than the intrauterine administration of G-CSF [318]. The use of intrauterine G-CSF was associated with significantly higher biochemical and clinical pregnancy rates among women with a thin endometrium or repeated IVF failures in comparison with no treatment or a placebo [319]. More clinical trials are required to ascertain the role of G-CSF in RIF and RPL. 

### 9.6. Tumor Necrosis Factor (TNF)-α Inhibitors

TNF inhibitors work by blocking TNF-α from binding to its receptors (TNFRI and TNFRII), thus suppressing the immune response [15]. These inhibitors also reduce the activity of transcription factors, proteases, and protein kinases (such as NF-κB, caspases, and MAPK) and decrease the release of pro-inflammatory cytokines, chemokines, and adhesion molecules. Additionally, they suppress the development of CD4+ T cells into Th1 and Th17 cells [15]. TNF-α inhibitors have been used to treat RPL to reduce the rate of immune rejection. Females with RPL who were treated with TNF inhibitors experienced better pregnancy outcomes. However, there are still insufficient data to fully support the use of TNF inhibitors in treating RPL [15].

A randomized controlled trial enrolling RPL patients (>3 abortions) with innate immune disorders reported that etanercept (a TNF inhibitor), 25 mg per week starting from the first day after menstruation, significantly reduced TNF-α and NK cell activity. Moreover, female patients treated with etanercept had a higher live birth rate than those treated with placebo [320]. In a prospective study of a single arm of patients with RIF, etanercept was associated with successful implantation in 75.9% of the cohort [314]. In another study, 62% of the cohort achieved a live birth or ongoing pregnancy; however, 56.7% of the live births were preterm (<37 weeks) and 60.5% were underweight (<2500 g) [321].

Adalimumab (another TNF-α inhibitor) and IVIGs significantly improved IVF outcomes in young infertile women with Th1/Th2 cytokine elevation [320,321]. Conversely, there was no significant difference between IVIG treatment alone [322,323]. Anti-TNF-α (adalimumab or certolizumab) has been used in refractory antiphospholipid syndrome with good obstetric results in 70% of patients [324]. Moreover, TNF-α blockers can be safely used during implantation and pregnancy [324]. Anti-TNFα is probably suitable only in RPL patients with autoimmune diseases who respond well to the therapy under normal conditions. 

### 9.7. Allogenic Peripheral Blood Mononuclear Cell (PBMC) Immunotherapy

PBMC therapy or lymphocyte immunotherapy (LIT) consists of collecting peripheral blood mononuclear cells from the husband or a third party and injecting them intradermically (in the forearm or thigh) into the prospective mother to prepare the immune system to tolerate the embryo’s antigens [319,320]. Various mechanisms have been suggested for the effectiveness of LIT, such as enhancing the expression of anti-paternal cytotoxic antibodies (APCAs), progesterone-induced blocking factor (PIBF), anti-idiotypic antibodies (Ab2), and mixed lymphocyte reaction blocking antibodies (MLR-Bf), as well as a reduction in the Th1/Th2 ratio and a deviation in the pattern of cytokine production [325]. Allogeneic PBMC therapy could enhance the percentage of CD4+ CD25+ Treg cells [326] and shift the balance of Th1/Th2 toward Th2 immunity in peripheral blood, which favors pregnancy. In addition, PBMC therapy significantly reduces the frequencies of Th17 and NK cells while enhancing the frequency of Treg cells. PBMC therapy can substantially modulate the maternal immune system by improving the Treg/Th17 paradigm and regulating the expression of Treg and Th17 cell-associated cytokines, transcription factors, and miRNAs. This treatment can also increase the live birth rate in RPL patients [327]. 

In a prospective study, LIT improved the pregnancy and live birth rates in RPL patients [328]. In another retrospective analysis, the live birth rate was significantly higher in the LIT group with RPL compared to no therapy [329]. A retrospective analysis of a multicenter, observational study that enrolled 1096 couples with a history of two or more spontaneous miscarriages showed higher gestation success in the LIT group (60.1% vs. 33.1%; *p* < 0.001) [301]. In another study with RPL patients, the abortion rate was significantly lower in the LIT group than in the control group, which only received progesterone [330]. An investigation showed the effectiveness of LIT in primary but not secondary RPL patients [331]. On the other hand, paternal lymphocytes were more effective than third-party lymphocytes in RPL patients [332].

The REMIS study, a double-blind, multicenter, randomized clinical trial, showed that immunization with paternal PBMC did not improve pregnancy outcomes in women with RPL. Still, this study used only one immunization, and most cells were injected intravenously (the less immunogenic route) [333]. Two meta-analyses did not find significant differences in patients who received paternal cell immunization [334,335]. However, another meta-analysis showed a significantly higher success rate in the allogeneic PBMC immunotherapy group with RPL. Administration of the therapy before and during pregnancy dramatically improved the live birth rate in women with RPL and was superior to PBMC immunotherapy given only before pregnancy [336]. In a different meta-analysis, paternal cell immunization induced a significant difference in outcome compared to autologous vaccination, although the studies were small and at high risk of bias [337]. 

There is insufficient evidence to recommend LIT in patients with RIF. Possible complications, such as infections, autoimmune disorders, and irregular antibody formation, with LIT must be considered [19,338,339].

### 9.8. Intrauterine Peripheral Blood Mononuclear Cells

In patients with RIF, the implantation rate was significantly higher when they received intrauterine administration of autologous PBMCs (a mix of T and B lymphocytes and monocytes) activated by human chorionic gonadotropin (hCG) in vitro (23.66% vs. 11.43% in the control group) [340]. Similar results were observed in a study by Li et al. [341]. Implantation, clinical pregnancy, and live birth rates were significantly higher in women with four or more implantation failures compared to the control group (22.00% vs. 4.88%, 39.58% vs. 14.29%, and 33.33% vs. 9.58%, respectively) [340]. 

Various meta-analyses showed that intrauterine autologous PBMC infusion benefits clinical pregnancy and life birth rates [14,315,342,343,344]. However, other meta-analyses did not demonstrate an association between administering PBMCs into the uterine cavity before fresh or frozen-thawed embryo transfer and live birth rates in women with RIF [343]. 

In a retrospective study, women with RPL and low endometrial FoxP3+ Tregs received intrauterine Tregs infusion. Patients in the Tregs group had a higher live birth rate and lower miscarriage rate than women who did not have intrauterine Tregs infusion [345]. 

### 9.9. Intrauterine Autologous Platelet-Rich Plasma (PRP)

Intrauterine platelet-rich plasma (PRP) treatment may improve pregnancy outcomes in patients with RIF. In a retrospective study by Ban Y et al. [346], it was found that the β-hCG-positive rate, clinical pregnancy rate, and live birth rate were higher in the PRP group compared to the control group. A meta-analysis that included seven randomized control trials (with 861 patients experiencing thin endometrium, implantation issues, or pregnancy failure) also showed that women who received PRP infusion had significantly higher rates of clinical pregnancy, chemical pregnancy, live birth, and implantation compared to the control group [346]. However, there was no significant difference in miscarriage rate [346]. Two other meta-analyses also found that PRP could significantly increase the live birth rate in patients with RIF compared to blank and placebo groups [347,348]. In a recent clinical trial, intrauterine PRP was superior to intrauterine G-CSF in patients with RIF [349].

### 9.10. Lipid Emulsion (Intralipid) Intravenous Therapy

Evidence supports the administration of intralipid (parenteral fat emulsion containing soybean oil, glycerin, and egg phospholipids) in certain patients with RPL where standard treatments have failed [350]. Intralipid therapy is effective in suppressing in vivo abnormal NK cell function. It usually consists of a solution combining 4 mL of intralipid at a 20% dilution with 250 mL of saline solution. The effects of this therapy on the function and number of NK cells take up to 6 weeks [351]. In a single-blinded randomized controlled trial, the use of intralipid therapy in patients with previously failed IVF compared to controls was associated with significant increases in the biochemical pregnancy rate (40.38% vs. 16%) and take-home baby rate (28.8% vs. 10%) [352]. Moreover, a double-blind, randomized controlled trial showed that intralipid administration in women with unexplained RPL and positive NK cell activity undergoing IVF/ICSI cycles increased both the ongoing pregnancy rate and the live birth rate [352]. However, in another study, intralipid administration was associated with non-significant increases in the chemical pregnancy rate and the clinical pregnancy rate and a non-significant reduction in the spontaneous abortion rate [353].

A meta-analysis of five randomized controlled trials (RCTs) including 840 patients (3 RCTs: women with repeated implantation failure, 1 RCT: women with recurrent spontaneous abortion, 1 RCT: women who had experienced implantation failure more than once) showed that intralipid administration significantly improved the clinical pregnancy rate, ongoing pregnancy rate, and live birth rate in comparison to controls [354]. However, intralipid therapy had no beneficial effect on the miscarriage rate [354]. In another meta-analysis of twelve studies, intralipid administration in patients with RPL or RIF improved the implantation ratio, pregnancy rate, and live birth rate, with a reduction in miscarriage [354]. The meta-analysis of Rimmer et al. [355], which evaluated 843 women with RIF, included five randomized trials with a moderate risk of bias. The intralipid group had a higher chance of clinical pregnancy and live birth compared to no intervention [356]. In a more recent meta-analysis that included randomized control trials, intralipid increased the clinical pregnancy, ongoing pregnancy, and live birth rates in women with RPL or RIF compared to the control group. However, there was no difference in the miscarriage rate [356]. Intralipid treatment was effective in patients with RIF and RPL with elevated Th1 cells in their endometrial biopsy [357]. In a retrospective study with historical control, intralipid therapy did not improve the live birth rate and was not cost-effective in RPL or RIF patients with elevated NK cells [358]. Intralipid treatment may be only effective in a well-defined subgroup of patients [359].

### 9.11. Omega 3 Fatty Acid Supplementation

Supplementation with omega-3 fatty acids was successfully used in RPL patients with antiphospholipid syndrome [360]. As described by Mu and coworkers [361] in a recent review, the rationale behind the use of omega-3 fatty acids was to decrease the formation of radicals and decrease the proinflammatory lipid products with a concomitant increase in resolvins, which in turn would modulate immune cells to a tolerogenic response. In addition, the use of omega-3 fatty acids modulated the gut microbiota and the production of metabolites, which decreased the general proinflammatory response observed in RPL patients [26]. Canela and coworkers [362] analyzed the phospholipids of the lipid emulsion-treated patients and concluded that significant changes were observed in patients with RIF and RPL. These changes can be used as biomarkers. More clinical trials are required to determine the importance of this treatment in RPL and RIF. 

### 9.12. Low-Molecular-Weight Heparin (LMWH)

International professional guidelines recommend heparin treatment for antiphospholipid syndrome [363,364]. Combining heparin plus aspirin during pregnancy may increase the live birth rate in women with persistent antiphospholipid antibodies and RPL compared to the aspirin treatment alone [365,366].

Several studies have found that LMWH is associated with increased chances of live birth in women with thrombophilia and pregnancy loss [367,368]. A randomized study of women with RPL and negative antiphospholipid antibodies showed a significantly higher take-home baby rate in the LMWH group compared to the control group [369]. A meta-analysis of 8 randomized control trials also demonstrated that LMWH significantly improved the live birth rate and reduced the miscarriage rate in patients with RPL compared to the control group [370]. Similarly, another meta-analysis, including RPL patients, showed that the number of live births was significantly higher in the group treated with LMWH and aspirin than in the group treated with aspirin alone [371]. However, a meta-analysis comparing LMWH with no LMWH during pregnancy in women with inherited thrombophilia and heterogeneous pregnancy morbidity did not show a significant difference in live birth rates with the use of LMWH [372]. Likewise, a meta-analysis did not demonstrate the beneficial effect of heparin, aspirin, or both on the live birth rate in patients with a history of RPL [373], and another randomized control trial showed that daily LMWH injections did not increase ongoing pregnancy or the live birth rate in women with unexplained RPL [374]. In patients with RPL and factor V mutation (Leiden), low-dose aspirin alone, LMWH plus aspirin, or LMWH alone had comparable live birth rates [375].

A meta-analysis that included three small randomized control trials showed no differences in the live birth rate, miscarriage rate, gestational age, or birth weight between patients with RPL who received heparin and patients without treatment [376]. In another meta-analysis involving women with unexplained RPL (5 studies, 1452 participants), LMWH reduced the risk of miscarriage in women suffering ≥ 3 miscarriages. Still, no substantial influence was found on the live birth rate, preterm birth, preeclampsia, or small for gestational age [377]. A recent meta-analysis, including studies with RPL and using LMWH with or without low-dose aspirin, did not demonstrate benefits in live birth rates [378]. This analysis contrasts with the previous report of the same group in a retrospective study; the use of heparin reduced the rates of miscarriage in patients with unexplained RPL and patients with antiphospholipid syndrome or thrombophilia [379]. 

The ALIFE2 trial, a prospective randomized study that included 326 patients with inherited thrombophilia and RPL, did not find a difference in the live birth rate between patients treated with LMWH and controls (72% vs 71%) [380]. Thus, it is necessary to analyze the reasons for the discrepancies in all of these trials. 

### 9.13. Low-Dose Acetylsalicylic Acid 

Low-dose aspirin and heparin are indicated for treating antiphospholipid syndrome [381]. Aspirin alone induced a lower live birth rate than LMWH administered with aspirin in patients with RPL and antiphospholipid syndrome [381]. In the OPTIMUM treatment strategy, RPL or RIF patients with thrombophilia (altered lupus anticoagulant, anticardiolipin antibody, anti-β2-GP1 antibody levels, protein C and S activities, and factor XII levels) received 81 mg/day of aspirin with no heparin [279,382,383,384]. In patients with RPL, the live birth rate was 77.1% in the group treated with low-dose aspirin alone compared to 78% for those who received LMWH [384]. 

In a randomized study in patients with RPL without thrombophilia, low-dose aspirin (100 mg/day) resulted in the same live birth rate as enoxaparin (40 mg/day). In primary RPL (women who have never given birth to a live infant), 94% of pregnancies treated with enoxaparin resulted in live births, compared to 81% treated with aspirin [385]. Nami and coworkers reported [386] that in patients with one or two previous pregnancy losses, aspirin led to more human chorionic gonadotropin-detected pregnancies, fewer pregnancy losses, and more live births compared to a placebo. 

On the other hand, Mumford et al. reported that in women with a history of one to two prior losses, the administration of low-dose aspirin before conception did not show a significant difference in the abortion rate compared to a placebo [387]. Aspirin did not prevent recurrent miscarriage in women with at least three consecutive miscarriages in the first trimester. In this trial, the live birth rates were high in the aspirin and placebo groups (83.0% and 85.5%, respectively) [388].

Aspirin monotherapy cannot be considered for patients with RPL and RIF and a possible subclinical autoimmune or thrombophilia component. 

### 9.14. Vitamin D 

An in vitro study demonstrated that vitamin D therapy regulates T helper cell populations by inhibiting cytotoxic Th1 cell proliferation, promoting Th2 cells, suppressing Th17, and inducing Treg cells [389]. Also, vitamin D has immune regulatory effects on NK cell cytotoxicity, cytokine secretion, and degranulation process [390].

Vitamin D deficiency and insufficiency are associated with miscarriage [391], and 64.6% of individuals with RPL also had vitamin D insufficiency or deficiency [392]. Vitamin D supplementation is recommended in obstetric antiphospholipid syndrome [324]. Strangely, a meta-analysis concluded that whether preconception treatment of vitamin D deficiency protects against pregnancy loss in women at risk of miscarriage remains unknown [393]. In patients with RPL, the prevalence of aPL antibodies, ANAs, anti-ssDNA, and thyroperoxidase antibodies was significantly higher in those with low vitamin D levels than in those with normal levels [393]. 

Since vitamin D has been shown to regulate immune cell responses, it is unsurprising that its deficiency is involved in RPL and RIF. More well-designed trials should focus on the possible deficiency of vitamin D. 

### 9.15. Progesterone

Progesterone is an immunosuppressive hormone that can regulate NK cell activity and cytokine balance during trophoblast invasion and lead to expansion of the CD56bright population. Progesterone-induced blocking factor (PIBF) by lymphocytes expressing progesterone receptors and trophoblast cells shifts the balance to a Th2-type immune response [394]. Progesterone effectively suppresses the mTOR pathway in generating Th1 and Th17 cells and induces Treg cell differentiation [394,395,396]. A Cochrane meta-analysis demonstrated the benefit of progesterone for reducing recurrent miscarriage risk in women [397]. Another meta-analysis suggested that progesterone or similar molecules made little to no difference to the live birth rate of women with threatened or recurrent miscarriages. However, in the same meta-analysis, vaginal micronized progesterone may have increased live birth rates in women with a history of one or more previous miscarriages and early pregnancy bleeding [398]. In a recent meta-analysis, progesterone in women at increased risk of pregnancy loss probably increased live birth rates. In patients with threatened miscarriage, this therapy was more effective if there was a history of previous abortions [399]. Progesterone was more successful when administered during the luteal phase in RPL patients [338].

Even though progesterone has been used in the clinic for many years, well-designed clinical trials should define the best pharmacological combination to increase the fertility rate and pregnancy success. 

### 9.16. Intrauterine Human Chorionic Gonadotropin (hCG) Infusion

In a meta-analysis, clinical pregnancy rates but not live birth rates were significantly better in the intrauterine hCG infusion groups than in the blank and placebo groups [400]. In another meta-analysis, in women who experienced two or more implantation failures, the clinical pregnancy and live birth rates were significantly improved in the hCG group compared to the control group [401]. In a prospective double blind randomized clinical trial, intrauterine GCSF administration simultaneously with hCG injection showed light, but not significant improvement in pregnancy rate [402].

The use of hGC is still preliminary, and several alternative routes should probably be used to validate its effects.

### 9.17. Anti-Obesity Drugs to Increase Fertility

The increase in overweight and obesity incidence in recent years may have significant consequences on fertility rates. Obesity has been shown to have a negative impact on endometrial receptivity, modifying the window of implantation [403,404]. It has been postulated that the link between obesity and subclinical inflammation, as described in metabolic syndrome, is responsible for the high rates of implantation failure and recurrent pregnancy loss in obese women [405]. Therefore, since metabolic changes like insulin resistance are associated with an array of immune and endocrine responses, the use of treatments to decrease obesity and insulin resistance may increase the fertility rate and decrease recurrent abortion incidence. 

Metformin has been used to treat women with polycystic ovary syndrome (PCOS), who have higher rates of RIF and RPL [406]. The drug has also been used to treat gestational diabetes and seems to benefit other pregnancy complications in obese women [407,408]. It is assumed that metformin, besides decreasing insulin resistance, modulates the immune response, which may affect the adipose tissue response and adipokine secretion and function. 

Recently, glucagon-like peptide-1 receptor agonists (GLP-1a) have been used to treat diabetes and obesity [409]. It has been proposed that decreasing adipose tissue increases fertility [409,410]. However, well-designed clinical trials are needed to determine the effectiveness of the treatment before programmed pregnancy or IVF procedures.

The ESHRE guidelines for the different therapies analyzed are as follows:

1. Glucocorticoids are not recommended for treating unexplained RPL or RPL exhibiting specific immunological biomarkers. There is insufficient evidence to endorse the use of progesterone for enhancing live birth rates in women with RPL and luteal phase insufficiency. However, vaginal progesterone may have a positive impact on live birth rates for women with three or more pregnancy losses combined with vaginal bleeding in subsequent pregnancies.
2. The use of heparin or low-dose aspirin is not advised in RPL patients without antiphospholipid syndrome, as evidence indicates that these interventions do not improve live birth rates in women with unexplained RPL.
3. There is also insufficient evidence for the effectiveness of human hCG in improving live birth rates among women with RPL and luteal phase insufficiency. Additionally, there is inadequate support for the use of metformin supplementation during pregnancy to prevent pregnancy loss in women with RPL and glucose metabolism anomalies.
4. Counseling women with RPL about the general recommendation to consider prophylactic vitamin D supplementation before conception may be beneficial. Low-dose folic acid is routinely initiated preconceptionally to prevent neural tube defects; however, it has not been demonstrated to avoid pregnancy loss in women with unexplained RPL. Due to inconclusive evidence, current guidelines neither endorse nor recommend using vitamin supplements as treatment. Patients should receive appropriate advice regarding the potential harms of vitamin supplements, notably vitamins E and A.
5. No evidence supports the recommendation of G-CSF in unexplained RPL.
6. Lymphocyte immunization therapy is not advised to treat unexplained RPL due to its lack of significant efficacy and potential for serious adverse effects. However, the administration of repeated and high doses of IVIGs early in pregnancy may increase live birth rates in women who have experienced four or more instances of unexplained RPL.
7. There is insufficient evidence to support intralipid therapy as a means of improving live birth rates in women with unexplained RPL.
8. According to the European Society of Human Reproduction and Embryology (ESHE), substantial studies on alternative therapies for couples experiencing RPL, including homeopathy, bioresonance therapy, and NaPro technology, are lacking.

**Table 2:** Summary of the different treatments used in RIF and RPL.

Treatment	Rationale	Effect	References
Corticosteroids (Treatment Level I)	Decrease in peripheral NK cells and increase tolerogenic activity. Combined with aspirin in patients with autoimmune antibodies.	Decreased cytotoxic function.	[244,245,249]
		No suppressive effect	[246]
		Increased implantation rate in IVF.	[250,251]
		Increased implantation rate and pregnancy success.	[253,254]
		No increase in live birth rates.	[255]
	Combined with aspirin and heparin in antiphospholipid syndrome.	Increased implantation and pregnancy success.	[256,257,258,259,260]
Hydroxy- Chloroquine (Treatment Level I)	Anti-thrombotic and immunomodulatory properties.	Decreased pregnancy loss.	[261,262,263,264,265]
		Effect dependent on dose.	[265,266]
	Combined with conventional treatment in antiphospholipid syndrome.	Enhanced Tregs, diminished Th17.	[267]
		Does not prevent further miscarriage.	[268]
Calcineurin inhibitors (Treatment Level II)	Cyclosporine and Tacrolimus. Immunosuppressive agents with risk of birth defects [264,265].	Increased implantation and pregnancy rate.	[272,273,274,275]
		Hypertensive disorders with treatment	[276]
		No increase in implantation rate.	[277]
		Increased implantation success and pregnancy outcome.	[278,279,280]
	Low-dose tacrolimus in women with immune disorders alone or combined with heparin. Low side effects.	Decreased Th1/Th2 ratio.	[281,282]
		Risk-benefit effect in endometriosis	[283,284]
	Sirolimus (rapamycin) inhibits the mTOR pathway that is altered in some RIF and RPL patients [279,280]	Phase II clinical in altered Th17/Treg patients. Increased implantation and pregnancy success.	[285]
Intravenous immunoglobulins (Treatment Level I)	Inhibition of HLA antibodies decreases Fc receptor expression and modulates NK cells.	Increased pregnancy success. Better efficiency at high doses.	[286,287,288,289,290,291,292,293,294,295,296]
		Effective in women with immunological problems	[297,298,299,300,301,302,303,304,305,306,309,310]
Granulocyte colony-stimulating factor (G-CSF) (Treatment Level II)	Tolerogenic response. Increase in Tregs/IL-10 [305,306].	Increased pregnancy success.	[313]
		There is no difference compared to placebo.	[314]
		Subcutaneous injections have a better effect on women’s ongoing procedures.	[315]
		Subcutaneous G-CSF increased implantation success in RIF patients.	[316,317,318,319]
Anti-TNFα (Treatment Level II)	Inhibition of TNFα decreases local inflammatory milieu.	Benefit for RPL and RIF patients with autoimmune spectrum.	[320,321]
		Combined with IVIG, it increased pregnancy success.	[324]
Allogenic peripheral blood mononuclear cell (PBMC) immunotherapy. (Treatment level II)	Generation of tolerogenic response to HLA antigens from the father and fetus [319,320,321].	Increased successful pregnancies in some trials.	[328,329,330,333,334,336,337]
		Benefit in primary RPL only.	[331]
		No beneficial effect.	[334,335]
		Therapy may have complications.	[19,338,339]
Autologous Intrauterine (PBMC) (Treatment Level II)	PBMC is activated by human chorionic gonadotropin to generate a local tolerogenic response.	Increased successful pregnancies in RPL patients.	[340,341,342,343,344]
		Increased Tregs in patients with low endometrial Treg.	[345]
Intrauterine autologous platelet-rich plasma (PRP) (Treatment Level II)	Decrease in local inflammatory response.	No significant effects.	[346]
		Improved live pregnancies in RIF patients.	[347,348]
		PRP therapy was superior to G-CSF infusion.	[349]
Intralipid/Intravenous lipid emulsions (treatment Level II)	Suppression of NK cytotoxic function [344,345] and probably T CD8 cells.	Increased pregnancy rate in previously failed IVF.	[350,352,355]
		No effect on pregnancy rate.	[353,354,356]
		Effective in patients with high Th1 in endometrial biopsy.	[357]
		No effect in patients with high endometrial NK cells	[359]
Omega-3 fatty acid oral supplementation (Treatment Level II)	Decreases peroxide formation—generation of resolvins to decrease the inflammatory response.	Positive effect in antiphospholipid syndrome RPL patients with conventional treatment.	[360,361,362]
Low molecular weight heparin (LMWH). (Treatment Level IV)	Decreases thrombotic risk in patients with antiphospholipid syndrome. Used as a guideline for antiphospholipid patients [357,358].	Increased live birth rate in RPL patients with persistent antiphospholipid antibodies.	[365,366,369,370,371]
		Increased live birth rates in patients with thrombophilia and RPL.	[367,368,379,380]
		There are no significant differences in patients with inherited thrombophilia and heterogeneous pregnancy morbidity. No beneficial effects.	[372,376,377,378]
Low-dose acetylsalicylic acid. (Treatment Level IV)	A co-treatment in antiphospholipid syndrome.	Combination treatment with LMWH enhanced birth rates compared to aspirin monotherapy.	[381,382,383,384,385,386]
		Low success rate with monotherapy	[387,388]
Vitamin D (Treatment Level II)	Deficiency in vitamin D is related to impaired immune response. Decreases the Th17 cell population	Vitamin D deficiency is observed in RPL patients.	[391]
		Decreased vitamin D in antiphospholipid syndrome	[324,393]
Progesterone (Treatment Level I)	Decreases the inflammatory response—decreases macrophages, NKs, and T cell activation [388,389,390]. Suppresses mTOR pathway.	Increased pregnancy rate (vaginal).	[397,399]
		No effect.	[398]
Intrauterine human chorionic gonadotropin (hCG) (Treatment Level II)	Induces tolerogenic milieu	Increased fertility rate, but not live birth rate.	[400,401]
		Lower effect than GM-CSF	[401]
		Intrauterine GCSF administration simultaneously with hCG injection may increase pregnancy outcome	[402]
Anti-obesity drugs (Treatment Level V)	Obesity decreases fertility rates. Subclinical inflammation may be responsible for reduced implantation rate and pregnancy success [403,404,405].	Metformin increases pregnancy success in polycystic ovary syndrome patients.	[406,407,408]

Table legend: The table summarizes the different therapies described in the literature. The treatment levels follow the Nursing-Johns Hopkins Evidence-Based Practice Model [411]. Level I is based on experimental study, level II is based on quasi-experimental study, level III is based on non-experimental study, level IV is based on opinion of expert societies, and level V is based on experiential and non-research evidence.

## 10. Future Perspectives

There is a growing necessity to thoroughly comprehend the physiological and pathophysiological processes associated with RIF and RPL, both primary and secondary. Recent advancements in reproductive medicine, particularly concerning the modulation of the adipose tissue response and adipokines, may play a pivotal role in identifying patients affected by these conditions. Furthermore, new insights into endometriosis and endometritis are likely significant factors in both RIF and RPL. Implementing innovative strategies to reduce the inflammatory burden within the endometrium may enhance current therapeutic options for these conditions. As highlighted in the review, the array of approaches has been varied, leading to complex and often challenging interpretations of the results.

Also, specific guidelines are needed to analyze and treat patients without a clear spectrum of autoimmune disorders. General progress has been made for patients with known autoimmune conditions, and good immunological screening and individualized use of immunomodulating therapy can probably be useful for RIF and RPL.

Microbiota analysis should be performed routinely in patients who attend fertility clinics since the presence of dysbiosis has been associated with decreased implantation and fetal survival. In addition, molecular mimicry of pathogens can be crucial in developing the autoimmune spectrum. 

## 11. Conclusions

Alterations in NK cells, Treg cells, Th2, and cytokines play major immunological roles in RPL and RIF. Therapies that correct NK cell disorders, inhibit Th17 and Th1 patterns, and promote Tregs and Th2 lymphocytes may improve live birth rates. 

RPL and RIF are complex conditions with multifactorial etiologies. Patients are a heterogeneous group with diverse immunological and non-immunological factors. Patients should be better classified depending on their immunological and endocrinological factors to design treatment approaches and achieve positive outcomes. In summary, individualized therapy should be considered.

Since infectious diseases and microbiota dysbiosis are increasing, medical screenings considering both factors are suggested. 

**Figure 1:**
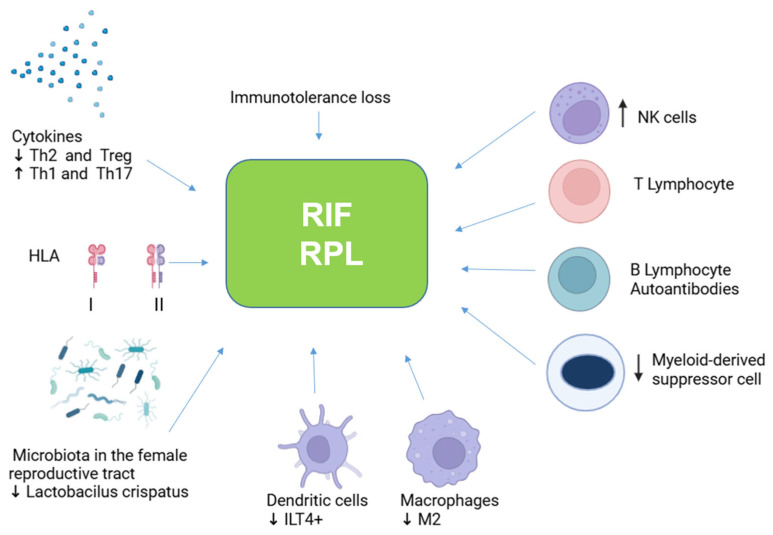
The figure illustrates the major elements studied in RIF and RPL.

Figure 1 summarizes the general point of the review.

## Data Availability

Not applicable.
